# Cystatin B Attenuates Cerebral Ischemia Reperfusion Injury by Inhibiting the JAK2/STAT3 Signaling Pathway

**DOI:** 10.1002/cns.70818

**Published:** 2026-03-29

**Authors:** Gang Zhou, Fengjiao Hu, Ju Gao, Lei Wang, Xiangbo Wu, Yu Tian, Peng Zhang, Guangming Xia, Feng Wan, Jinhua Wang

**Affiliations:** ^1^ Department of Neurology Huanggang Central Hospital of Yangtze University Huanggang China; ^2^ Medical Science Research Center, Zhongnan Hospital; Basic Medical School Wuhan University Wuhan China; ^3^ Basic Medical School Wuhan University Wuhan China

**Keywords:** cerebral ischemia reperfusion injury, cystatin B, JAK2/STAT3 pathway, stroke

## Abstract

**Background:**

Cerebral ischemia reperfusion injury (CIRI) poses a significant clinical and economic burden worldwide. Therefore, it is essential to identify key regulators that may improve stroke prognosis. Cystatin B (CSTB) is known to be involved in neuroprotection, inflammation modulation, and apoptosis regulation, but its specific function and mechanisms in CIRI remain unclear.

**Methods:**

We employed gain‐ and loss‐of‐function approaches in a mouse model of transient middle cerebral artery occlusion (t/MCAO) and in cultured neurons subjected to oxygen–glucose deprivation/reperfusion (OGD/R). The effects were evaluated primarily using a combination of quantitative PCR, Western blot, and immunofluorescence staining to assess neurological deficits, inflammatory responses, and apoptosis, as well as to elucidate the underlying mechanisms.

**Results:**

Our findings demonstrated that CSTB significantly attenuated CIRI, as evidenced by the mitigation of neurological deficits, inflammation, and apoptosis. Mechanistically, the protective effects of CSTB were associated with the suppression of the JAK2/STAT3 signaling pathway.

**Conclusion:**

This study identifies CSTB as a novel negative regulator of CIRI. Its protective role is mediated through the inhibition of apoptosis and inflammatory responses via the JAK2/STAT3 axis, suggesting its potential as a therapeutic target for ischemic stroke.

## Graphical abstract

Using experimental models of stroke, we demonstrate that Cystatin B (CSTB) alleviates brain injury following cerebral ischemia reperfusion. This protection is mediated by inhibiting the JAK2/STAT3 signaling pathway, which reduces neuroinflammation and neuronal apoptosis. Our findings highlight CSTB as a promising therapeutic target for ischemic stroke.

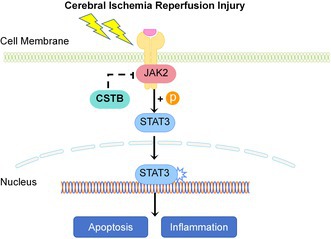

## 1 Introduction

Stroke remains a leading global cause of disability and mortality, accounting for 7.3 million deaths and 160.5 million disability‐adjusted life years (DALYs) globally in 2021, and poses a significant public health challenge due to its high incidence, recurrence rate, and functional impairment [1, 2]. Ischemic stroke, the predominant type of stroke, is an acute cerebrovascular disease caused by the blockage or severe stenosis of cerebral arteries, which leads to reduced blood flow, insufficient oxygen supply, and subsequent necrosis of brain tissue [3, 4, 5]. The restoration of cerebral blood flow through revascularization therapies, including intravenous thrombolysis and endovascular thrombectomy, represents the fundamental therapeutic approach for acute ischemic stroke [6, 7]. However, the process of reperfusion itself can paradoxically induce further cerebral damage, a pathophysiological phenomenon known as cerebral ischemia reperfusion injury (CIRI) [8, 9]. This secondary injury mechanism involves a complex cascade of molecular events including explosive oxidative stress, pervasive neuroinflammation, and the activation of multiple cell death pathways [3, 7, 10]. The sudden reintroduction of oxygen during reperfusion triggers the massive production of reactive oxygen species (ROS), resulting in mitochondrial dysfunction, lipid peroxidation, and DNA damage [3, 11]. Simultaneously, activated microglia and astrocytes release pro‐inflammatory cytokines, exacerbating blood–brain barrier disruption and neuronal apoptosis [12, 13]. Despite advancements in revascularization techniques, the clinical benefits of reperfusion therapies remain limited by narrow treatment time windows and the inherent risks of hemorrhagic transformation and CIRI [10, 14, 15]. This highlights the critical need for novel adjuvant neuroprotective strategies that can mitigate reperfusion injury and improve long‐term functional outcomes.

Cystatin B (CSTB), a member of the cystatin superfamily, is an intracellular thiol protease inhibitor that targets cysteine proteases including cathepsins L, H, and B [16]. It has been extensively studied in relation to genetic variations and their implications in neurological disorders, particularly progressive myoclonus epilepsy type 1 (EPM1) [17, 18]. Emerging genetic and clinical evidence now suggests an association between CSTB and the pathogenesis of CIRI. Transcriptomic and molecular analyses have revealed altered *Cstb* gene expression following CIRI, suggesting its potential role in modulating cellular responses during reperfusion [19, 20]. Previous studies have implicated CSTB in several mechanisms relevant to CIRI pathology, including neuroprotection [21, 22], inhibition of protease‐mediated damage [23, 24], regulation of autophagy [25], apoptosis [17], reactive oxygen species (ROS) [26] and inflammatory pathways [23, 27]. However, the specific mechanisms by which CSTB contributes to CIRI remain poorly understood and require further investigation.

In the present study, we observed an elevated expression of CSTB in mouse brain neurons 24 h after CIRI. Furthermore, knockdown of CSTB was associated with significantly aggravated CIRI‐induced neurological dysfunction, brain infarction, neuronal injury, inflammation, and apoptosis. In contrast, persistent overexpression of CSTB enhanced the neuroprotective effect against CIRI. Mechanistically, we provided experimental evidence that CSTB may reduce CIRI via down‐regulating the JAK2/STAT3 signaling pathway. Thus, our findings suggest that CSTB critically influences neuronal cell fate in response to CIRI, positioning it as a promising novel therapeutic target for ischemic stroke.

## 2 Materials and Methods

### 2.1 Bioinformatics Analysis of CSTB Expression

CSTB expression levels were analyzed using the NCBI datasets. Raw RNA‐seq reads were normalized by transcripts per million (TPM) transformation. Statistical significance of intergroup differences was assessed using the Student's *t*‐test, and results were visualized using box plots.

### 2.2 Construction of Cstb Knockdown and Overexpression Mice

To construct CSTB knockdown and overexpression models, male C57BL/6 mice (6–8 weeks old, 26–28 g) were housed in a specific pathogen‐free (SPF) conditions. The environment was maintained at 22°C–24°C with 40%–70% humidity and a 12 h light/dark cycle, with food and water available *ad libitum*.

To achieve AAV9‐mediated CSTB knockdown or overexpression in brain tissue via intracerebroventricular delivery, AAV9‐shCstb and AAV9‐Cstb were used, with AAV9‐shRNA (scramble sequence: CCTAAGGTTAAGTCGCCCTCG) and AAV9‐Vector serving as the respective controls. Mice were anesthetized with 2.0% isoflurane in an oxygen/nitrous oxide mixture. After scalp incision and exposure of the bregma, a needle was stereotaxically implanted 0.2–0.5 mm posterior to the bregma and 1.0 mm lateral to the midline at a depth of 2.5–3.0 mm. A viral suspension (2 μL, 1 × 10^13^ v.g./mL) was infused slowly into the target site. The needle was kept in place for 60 s before withdrawal. All subsequent experiments were conducted 3 weeks post‐injection.

### 2.3 Construction of CIRI Model and Grouping in Mice

C57BL/6 mice were randomly allocated into eight experimental groups (*n* = 18 per group) based on their genotype and intended intervention: AAV9‐shRNA Sham, AAV9‐shCstb Sham, AAV9‐shRNA t/MCAO, AAV9‐shCstb t/MCAO, AAV9‐Vector Sham, AAV9‐Cstb Sham, AAV9‐Vector t/MCAO, and AAV9‐Cstb t/MCAO.

Prior to surgery, mice were anesthetized with 2.0% isoflurane in an oxygen/nitrous oxide mixture. A longitudinal incision was made in the left cranial skin to expose the skull, and connective tissue was carefully removed. Regional cerebral blood flow (rCBF) was monitored using a laser‐Doppler flowmetry system (Periflux System 5010, Perimed) with a probe fixed to the skull (1.5 mm posterior and 3–4 mm lateral to the bregma). Rectal temperature was maintained at 37.0°C ± 0.5°C with a homeothermic heating pad. Transient middle cerebral artery occlusion (t/MCAO) was induced by introducing a 6–0 silicon‐coated nylon monofilament (602156PK10Re, Doccol) into the left external carotid artery, advancing it into the internal carotid artery, and positioning it to block the origin of the middle cerebral artery (MCA). Successful occlusion was defined by a > 75% reduction in rCBF. After 45 min of ischemia [28, 29], the filament was withdrawn. Reperfusion was confirmed when rCBF recovered to > 70% of baseline within 10 min. Sham‐operated mice were subjected to the same surgical procedures as the t/MCAO group, except for the induction of cerebral ischemia.

### 2.4 Neurological Function Assessment

Neurological function was assessed 24 h after reperfusion or sham surgery using the modified Longa scoring system (5‐point scale), as previously described [30].

### 2.5 2,3,5‐Triphenyl‐2H‐Tetrazolium Chloride (TTC) Staining

Following the neurological evaluation, mice were anesthetized with sodium pentobarbital (1%, 50 mg/kg, intraperitoneally) and euthanized. For TTC staining, brain tissues from six randomly selected mice per group were collected. The harvested brain tissue was frozen at −20°C for 30 min and sectioned coronally into 1‐mm slices. Typically yielding seven sections, the slices were promptly immersed in 2% TTC solution and incubated at 37°C for 10 min with periodic gentle agitation to ensure uniform staining. After fixation in 4% neutral paraformaldehyde solution, the infarct volume and edema ratio were quantified using Image‐Pro Plus 6.0 software (Media Cybernetics).

### 2.6 Pathological Analysis

Mouse brain tissues were harvested and immediately fixed in 10% neutral formalin, followed by dehydration, paraffin embedding, and sectioning at 5 μm. For histopathological assessment, sections were stained with hematoxylin and eosin (H&E; G1005, Servicebio) and examined under a light microscope (ML31, Mshot).

For CD11b immunofluorescence staining, sections underwent antigen retrieval with EDTA by high‐temperature heating for 20 min and were blocked with 10% goat serum. The sections were then incubated overnight at 4°C with an anti‐CD11b primary antibody (A26198, Abclonal). After washing three times with PBS, the sections were treated with a fluorescent secondary antibody (Alexa Fluor 568 goat anti‐rabbit IgG (H + L) A11036, Invitrogen) for 1 h. Nuclei were stained with DAPI. Apoptosis was evaluated using a TUNEL assay kit (C1090, Beyotime) according to the manufacturer's instructions. Nuclei were counterstained with DAPI. Changes in mitochondrial membrane potential, another key indicator of apoptosis, were detected with the JC‐1 kit (C2006, Beyotime) following the manufacturer's protocol. All fluorescent images were acquired using a fluorescence microscope (BX51, OLYMPUS) and analyzed with IPP software.

### 2.7 Generation of CSTB Knockdown and Overexpression Stable Cell Lines in HT22 Cells

The HT22 neuronal cell line was purchased from Procell (CL‐0679) and cultured in high‐glucose Dulbecco's Modified Eagle Medium (DMEM; PM150210, Procell) supplemented with 10% fetal bovine serum (FBS‐S500, NewZerum) and 1% antibiotic–antimycotic solution (PB180120, Procell) at 37°C in a 5% CO_2_ incubator.

To establish stable cell lines, a *Cstb*‐targeting shRNA (designated shCstb; a non‐targeting shRNA as the control) was constructed and ligated into the pLKO.1 vector. For overexpression, the mouse *Cstb* coding sequence was amplified with Phanta Max Super‐Fidelity DNA Polymerase (P505‐d1, Vazyme) and cloned into the pHAGE vector using the ClonExpress II one‐step kit (C112‐02, Vazyme) to generate the pHAGE‐CMV‐Cstb‐Flag‐PGK‐puro plasmid (designated Flag‐Cstb; empty vector served as Flag control). Both recombinant plasmids were then co‐transfected with the packaging plasmids pMD2.G and psPAX2 into HEK293T cells using polyethyleneimine (PEI; 23966‐1, Polysciences). 48 h after transfection, the viral supernatant was collected and filtered through a 0.22‐μm filter (SLGPR33RB, Millipore). The lentivirus supernatant was used to infect HT22 cells in the presence of 10 μg/mL polybrene (SC‐134220, Santa Cruz). Transduced HT22 cells were selected with 2.5 μg/mL puromycin (BLGE320‐25MG, Biolight) for 48 h. Finally, total protein was extracted from the resistant cells to evaluate CSTB expression, which confirmed the successful establishment of the stable cell lines.

### 2.8 Isolation, Culture, and Genetic Modification of Rat Primary Cortical Neurons

The use of rat primary cortical neurons is a common and reliable approach to model CIRI for mechanistic studies [31, 32]. Primary cortical neurons were isolated from neonatal (1–2 day‐old) Sprague–Dawley rats. The cerebral cortices were minced and digested with 0.25% trypsin (27250018, Gibco) for 15 min at 37°C. Digestion was terminated by adding DMEM/F12 medium (PB150315‐500, Procell) containing 10% fetal bovine serum (FBS‐CS500, Newzerum) and DNase (10104159001, Roche). The cell suspension was filtered through a 40‐μm strainer (G4211, Servicebio) to remove debris and centrifuged at 1500 rpm for 5 min at 4°C. The pellet was resuspended in DMEM/F12 medium. After cell counting and viability assessment, neurons were seeded onto poly‐L‐lysine (10 μg/mL, Sigma)‐coated dishes and maintained for 3 h in a 37°C, 5% CO_2_ incubator. The medium was then replaced with neurobasal medium (21103‐049, Invitrogen) containing 2% B27 (17504044, Gibco) and 1% penicillin–streptomycin. Cultures were maintained in the dark with medium changes every 48 h and used for experiments after 7 days in vitro.

For genetic manipulation of CSTB and JAK2 expression, adenoviral vectors were constructed. For CSTB overexpression, the coding sequence of Cstb was amplified from rat cDNA and cloned into pENTR‐U6‐CMV‐ATG‐flag‐T2A‐EGFP to generate the overexpression construct AdCstb (Ad‐Vector served as the control). To knock down CSTB or JAK2, shRNA sequences targeting CSTB or JAK2 were inserted into the pENTR‐U6‐CMV‐ATG‐flag‐T2A vector, generating the knockdown construct designated Ad‐shCstb and Ad‐shJAK2 (Ad‐shRNA served as the control). These entry plasmids were recombined with the adenoviral backbone plasmid pAd/PL‐DEST (V49420, Thermo Fisher) using Gateway LR Clonase II Enzyme Mix (2484478, Thermo Fisher). The recombinant constructs were linearized and transfected into HEK293A cells using polyethyleneimine (PEI; 24765‐100, Polysciences). Viruses were packaged with the Adeasy Adenovirus Packaging System (240009, Agilent Technologies), yielding a final titer of 1 × 10^10^ PFU/mL for subsequent neuronal transduction. The neurons were infected with an adenovirus at a multiplicity of infection (MOI) of 100.

### 2.9 Neuronal Oxygen–Glucose Deprivation/Reperfusion (OGD/R)

To establish an in vitro model of CIRI, neurons underwent OGD/R treatment. Briefly, primary neurons or HT22 cells were incubated in glucose‐ and pyruvate‐free DMEM (PM150271, Procell) under hypoxic conditions (94% N_2_, 5% CO_2_, 1% O_2_) for 3 h. Reperfusion was simulated by replacing the medium with normal Neurobasal medium and returning cultures to normoxic conditions for 6 h. Control cells were maintained under normoxic conditions throughout the experiment. To inhibit JAK2, cells were pre‐treated with 50 μM AG490 (HY‐12000, MCE) 1 h prior to OGD exposure.

### 2.10 Cell Viability and LDH Assays

Cell viability was assessed using a Cell Counting Kit‐8 (CCK‐8; C0039, Beyotime) according to the manufacturer's instructions. The release of lactate dehydrogenase (LDH) into the culture medium was measured using a colorimetric cytotoxicity detection kit (C0018S, Beyotime).

### 2.11 Quantification of Inflammatory Cytokines by ELISA

Following OGD/R stimulation, culture supernatants were collected. The levels of tumor necrosis factor‐alpha (TNF‐α; PT512 and PT516, Beyotime), interleukin‐6 (IL‐6; PI326 and PI328, Beyotime), and interleukin‐1β (IL‐1β; PI301 and PI303, Beyotime) were measured using commercial ELISA kits in accordance with the manufacturer's protocols.

### 2.12 Quantitative Real‐Time PCR (Q‐PCR)

Total RNA from tissues and cells was isolated using TRNzol reagent (DP424, TIANGEN). Complementary DNA was synthesized from the extracted RNA with a reverse transcription kit (TSK314M, TSINGKE). Q‐PCR was performed using ChamQ SYBR Master Mix (Q311‐03, Vazyme) on a LightCycler 480 system (Roche). β‐Actin served as the endogenous control. All primer sequences are provided in Table S1.

### 2.13 Western Blot Analysis

Cells or tissues were lysed in RIPA buffer (65 mM Tris–HCl pH 7.5, 150 mM NaCl, 1 mM EDTA, 1% Nonidet P‐40, 0.5% sodium deoxycholate, 0.1% SDS) containing protease inhibitors (4693132001, Roche) and phosphatase inhibitors (4906837001, Roche). Protein concentration was determined using a BCA assay kit (KTD3001, Abbkine). Equal amounts of protein were mixed with loading buffer and denatured at 95°C for 15 min. Samples were separated by 10% SDS‐PAGE and transferred to a 0.45‐μm PVDF membrane (IPVH00010, Millipore). After blocking with 5% skim milk for 1 h at room temperature, the membrane was incubated with the primary antibody overnight at 4°C. Following three washes with TBST, the membrane was probed with the appropriate secondary antibody. Signals were developed using an Omni‐ECL Ultra‐sensitive Chemiluminescent Detection Kit (SQ201, Epizyme) and detected using a Bio‐Rad ChemiDoc XRS+ imaging system. Antibodies used are listed in Table S2.

### 2.14 Coimmunoprecipitation (Co‐IP) Assay

The full‐length coding sequences (CDS) of *Cstb* and *JAK2* were amplified from human cDNA and subsequently cloned into the pcDNA3.1‐flag and pcDNA3.1‐HA vectors, respectively, yielding full‐length overexpression plasmids for Flag‐Cstb and HA‐JAK2. HEK293T cells were transfected individually or co‐transfected with the Flag‐Cstb and HA‐JAK2 plasmids. After 24 h, cells were lysed using IP lysis buffer. Following high‐speed centrifugation at 4°C, the protein‐containing supernatants were incubated overnight at 4°C with protein G agarose beads and the indicated tag antibodies for immunoprecipitation. The beads were then centrifuged at 3000 rpm at 4°C, washed three times with buffer containing 300 mM and 150 mM NaCl, resuspended in 2 × SDS loading buffer, and boiled at 95°C for 5–10 min. Finally, the samples were subjected to Western blot analysis.

### 2.15 Statistical Analysis

Data were analyzed using SPSS 21.0 and visualized with GraphPad Prism 8.0. Results are expressed as mean ± standard deviation (SD). Normality was assessed using the Shapiro–Wilk test. Differences between two groups were evaluated with the two‐tailed Student's *t*‐test for normally distributed data, or the Mann–Whitney *U* test for non‐normal data. Multiple‐group comparisons were performed using one‐way ANOVA followed by Bonferroni's post hoc test for homoscedastic data, or Tamhane's T2 test for heteroscedastic data. A *p* value less than 0.05 or 0.01 was considered statistically significant.

## 3 Results

### 3.1 CSTB Expression Is Upregulated After CIRI

**FIGURE 1:**
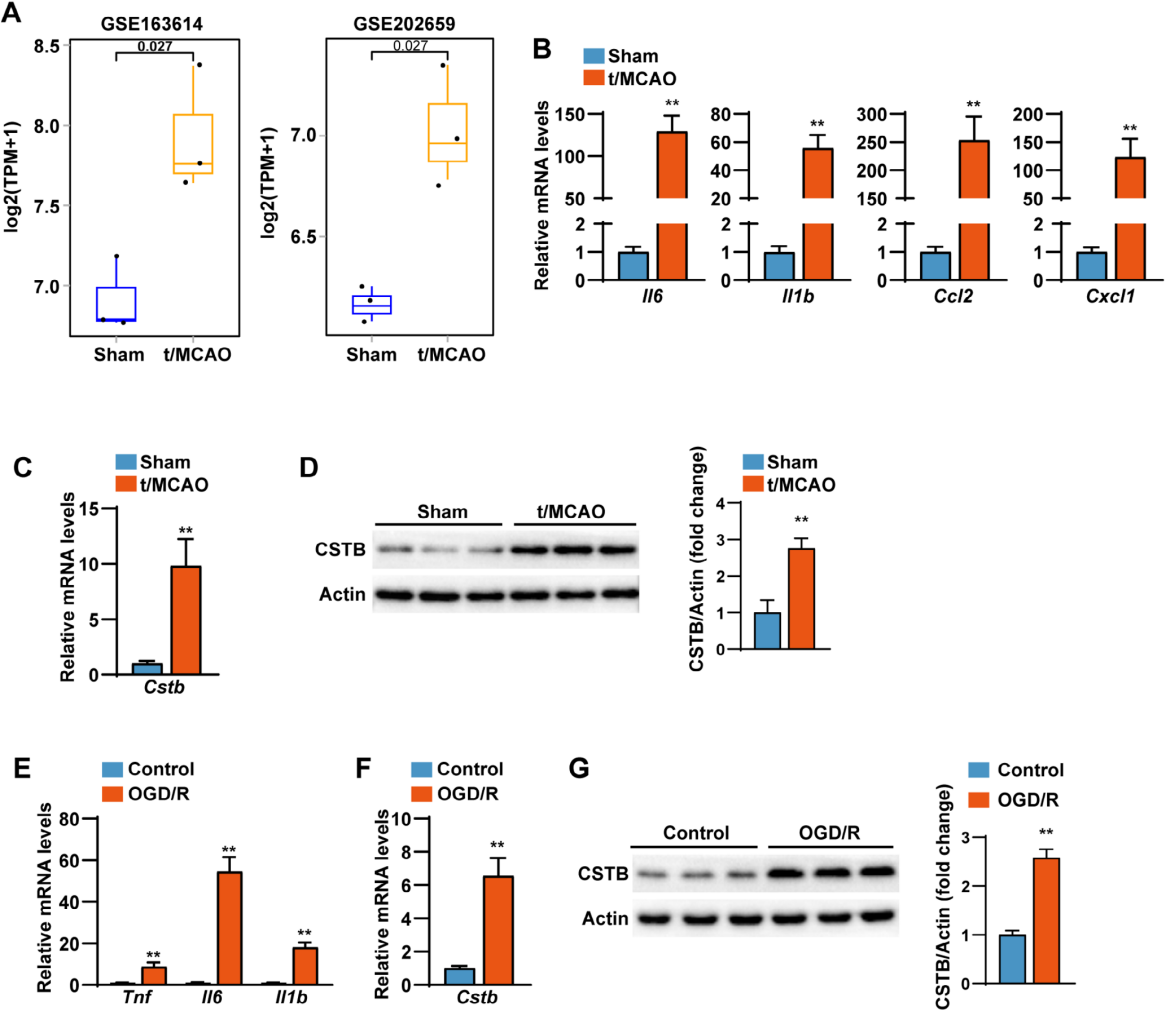
CSTB expression is upregulated after CIRI. (A) Comparative analysis of *Cstb* mRNA log2 fold change in mouse brain tissue from t/MCAO and Sham groups, based on data from NCBI datasets GSE163614 and GSE202659. (B) The mRNA levels of pro‐inflammatory cytokines (*Il6*, *Il1b*, *Ccl2* and *Cxcl1*) in brain tissues from t/MCAO and Sham groups (*n* = 4 per group). (C) Q‐PCR analysis results of the *Cstb* mRNA expression levels in mice brain tissues of t/MCAO and Sham groups (*n* = 4 per group). (D) Western blot (left) and quantification (right) results of CSTB protein expression levels in mice brain tissues of t/MCAO and Sham groups (*n* = 3 per group). (E) The mRNA levels of pro‐inflammatory cytokines (*Tnf*, *Il6* and *Il1b*) from OGD/R and Control groups. (*n* = 4 independent experiments). (F) Q‐PCR analysis results of the *Cstb* mRNA expression levels in OGD/R and Control groups (*n* = 4 independent experiments). (G) Western blot (left) and quantification (right) results of CSTB protein expression levels in OGD/R and Control groups (*n* = 3 independent experiments). Data are presented as the mean ± SD. *p < 0.05, **p < 0.01.

Analysis of the publicly available NCBI database (GSE163614, GSE202659) indicated a marked upregulation of *Cstb* mRNA expression in the t/MCAO group relative to sham‐operated mice (Figure 1A). To investigate the role of CSTB in CIRI, we established a stroke model by subjecting mice to 45 min of t/MCAO followed by 24 h of reperfusion. The mRNA levels of pro‐inflammatory cytokines (including *Il6*, *Il1b*, *Ccl2*, and *Cxcl1*) were significantly elevated in brain tissues from the t/MCAO group compared with the sham group (Figure 1B). Both Q‐PCR and Western blot analyses demonstrated that CSTB expression was significantly increased at the mRNA and protein levels in brain tissues 24 h after CIRI (Figure 1C,D).

To further validate these findings in vitro, rat primary cerebral cortical neurons underwent OGD/R (3 h/6 h). Neurons exposed to OGD/R exhibited substantially increased mRNA expression of inflammatory cytokines (*Tnf*, *Il6*, *Il1b*) compared with control cells (Figure 1E). Consistent with the in vivo data, CSTB expression was also significantly elevated in OGD/R‐treated neurons relative to controls (Figure 1F,G).

These findings suggest that CSTB may be involved in the pathological process following cerebral ischemic stroke.

### 3.2 CSTB Knockdown Aggravates CIRI in Mice

**FIGURE 2:**
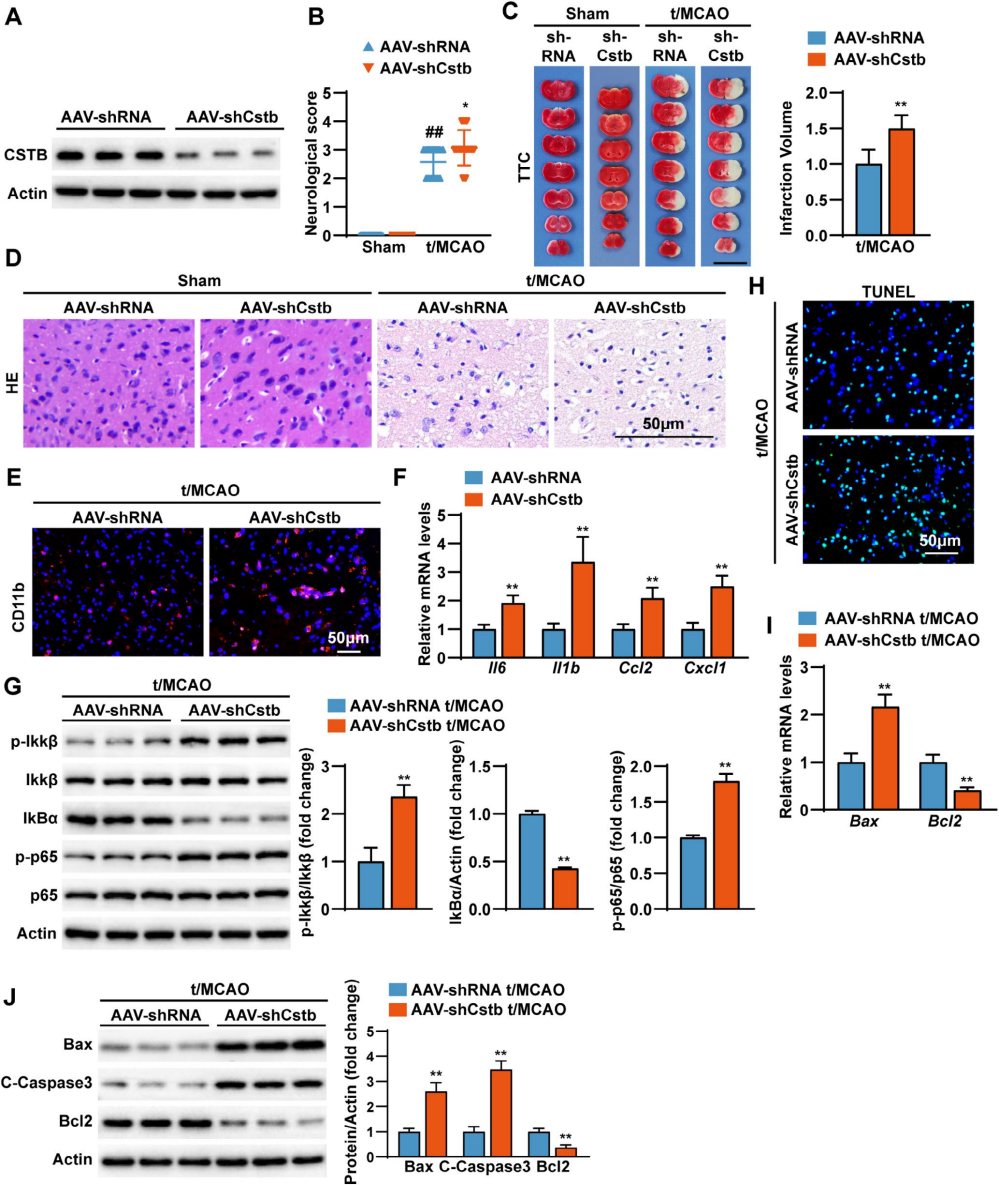
CSTB knockdown aggravates CIRI in mice. (A) Validation of *Cstb* knockdown in mouse brain tissue by Western blot analysis (*n* = 3 per group). (B) Neurological deficit scores in *Cstb*‐knockdown (AAV‐shCstb) and control (AAV‐shRNA) mice at 24 h after Sham or t/MCAO treatment (*n* = 14 per group). (C) Representative TTC‐stained brain sections (left) and quantitative analysis of infarct volume (right) in AAV‐shCstb and AAV‐shRNA mice following Sham or t/MCAO surgery (*n* = 6 per group). Scale bar, 10 mm. (D) Representative images of H&E‐stained cerebral cortex sections from AAV‐shCstb and AAV‐shRNA mice following Sham or t/MCAO surgery (*n* = 6 per group). Scale bar, 50 μm. (E) Representative immunofluorescence images of cortical brain sections from AAV‐shCstb and AAV‐shRNA mice following t/MCAO, co‐stained for CD11b (microglia, red) and DAPI (nuclei, blue) (*n* = 3 per group). Scale bar, 50 μm. (F) Q‐PCR analysis results of the mRNA expression level of pro‐inflammatory cytokines (*Il6*, *Il1b*, *Ccl2* and *Cxcl1*) in brain tissues from AAV‐shCstb and AAV‐shRNA mice following t/MCAO (*n* = 4 per group). (G) Western blot (left) and quantification (right) results of key NF‐κB signaling proteins (p‐IκBα, IκBα, p‐IκBβ, IκBβ, p‐p65, p65) in brain tissues from AAV‐shCstb and AAV‐shRNA mice following t/MCAO (*n* = 3 per group). (H) Representative images of TUNEL (green) and DAPI (blue) staining in the cerebral cortex sections from AAV‐shCstb and AAV‐shRNA mice following t/MCAO (*n* = 3 per group). Scale bar, 50 μm. (I) Q‐PCR analysis results of the mRNA expression level of *Bax* and *BCl2* in brain tissues from AAV‐shCstb and AAV‐shRNA mice following t/MCAO (*n* = 4 per group). (J) Western blot (top) and quantification (bottom) results of key apoptosis proteins (Bax, C‐Caspase3 and Bcl2) in brain tissues from AAV‐shCstb and AAV‐shRNA mice following t/MCAO (*n* = 3 per group). Data are presented as the mean ± SD. *p < 0.05, **p < 0.01.

To investigate the role of CSTB in ischemic stroke, we employed a loss‐of‐function approach using Cstb knockdown mice (AAV‐shCstb), with mice injected with AAV encoding non‐targeting shRNA (AAV‐shRNA) serving as controls. Knockdown efficiency was confirmed by Western blot (Figure 2A). Mice were subjected to t/MCAO surgery for 45 min ischemia followed by 24 h reperfusion.

We found that CSTB knockdown was associated with profound exacerbation of brain injury and ischemic neuroinflammation. As shown in Figure 2B, no significant difference in neurological scores was observed between the Sham shRNA and Sham shCstb groups (both scored 0), indicating that shCstb alone did not impair baseline neural function. However, following reperfusion, CSTB‐knockdown mice exhibited significantly more severe neurological deficits after (Figure 2B) and a substantially larger cerebral infarct volume, as quantified from TTC‐stained sections (Figure 2C). Histopathological analysis via H&E staining further revealed exacerbated tissue damage, characterized by heightened neuronal necrosis and structural disintegration in the cerebral cortex of knockdown mice (Figure 2D). A multi‐assay approach demonstrated widespread neuroinflammation, as shown by increased CD11b expression (Figure 2E), a surge in pro‐inflammatory cytokines (Il6, Il1b, Ccl2, and Cxcl1) mRNA levels (Figure 2F), and activation of the key NF‐κB inflammatory signaling pathway (Figure 2G).

Given the reported significance of neuronal apoptosis in ischemic injury [3, 33], we performed TUNEL staining and examined apoptosis‐related markers via Q‐PCR and Western blot. Our results showed that CSTB knockdown elevated the number of TUNEL‐positive cells (Figure 2H) and increased expression of the pro‐apoptotic proteins Bax and Cleaved‐Caspase 3 (C‐Caspase 3), while decreased anti‐apoptotic Bcl2 (Figure 2I,J), suggesting its involvement in cell apoptosis during CIRI. These results demonstrate that CSTB knockdown worsens CIRI in vivo.

### 3.3 CSTB Overexpression Alleviates CIRI in Mice

**FIGURE 3:**
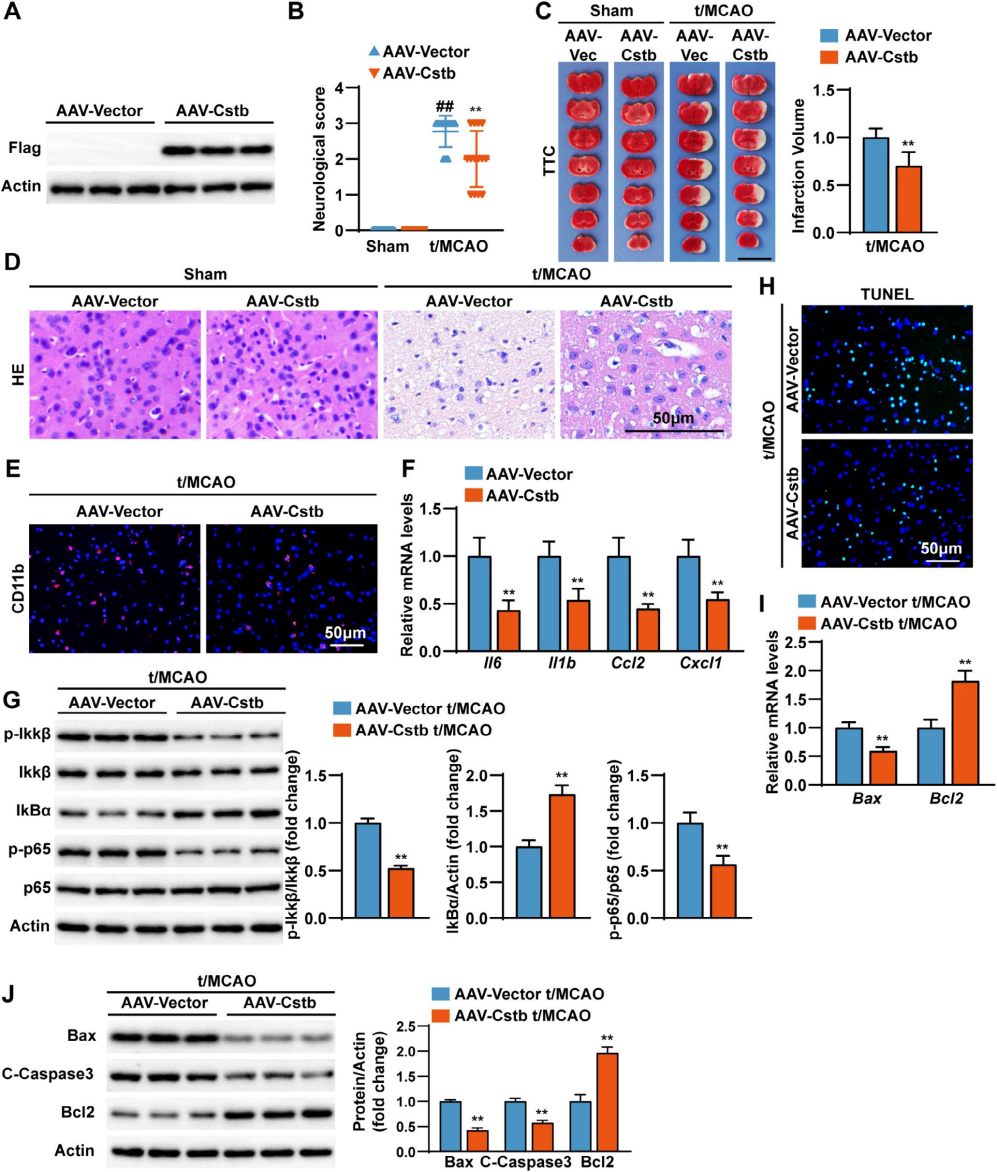
CSTB overexpression alleviates CIRI in mice. (A) Confirmation of successful *Cstb* overexpression in mouse brain tissue via Western blotting (*n* = 3 per group). (B) Assessment of neurological deficit scores in *Cstb*‐overexpression (AAV‐Cstb) and control (AAV‐Vector) mice at 24 h after Sham or t/MCAO treatment (*n* = 13–14 per group). (C) Representative TTC‐stained mouse brain sections (left) and quantitative analysis of infarct volume (right) in AAV‐Cstb and AAV‐Vector mice following Sham or t/MCAO surgery (*n* = 6 per group). Scale bar, 10 mm. (D) Representative images of H&E‐stained mouse cerebral cortex sections from AAV‐Cstb and AAV‐Vector mice following Sham or t/MCAO surgery (*n* = 6 per group). Scale bar, 50 μm. (E) Representative immunofluorescence images of mouse cortical brain sections from AAV‐Cstb and AAV‐Vector mice following t/MCAO, co‐stained for CD11b (microglia, red) and DAPI (nuclei, blue) (*n* = 3 per group). Scale bar, 50 μm. (F) Q‐PCR analysis results of the mRNA expression level of pro‐inflammatory cytokines (*Il6*, *Il1b*, *Ccl2* and *Cxcl1*) in brain tissues from AAV‐Cstb and AAV‐Vector mice following t/MCAO (*n* = 4 per group). (G) Western blot (left) and quantification (right) results of key NF‐κB signaling proteins (p‐IκBα, IκBα, p‐IκBβ, IκBβ, p‐p65, and p65) in brain tissues from AAV‐Cstb and AAV‐Vector mice following t/MCAO (*n* = 3 per group). (H) Representative images of TUNEL (green) and DAPI (blue) staining in the cerebral cortex sections from AAV‐Cstb and AAV‐Vector mice following t/MCAO (*n* = 3 per group). Scale bar, 50 μm. (I) Q‐PCR analysis results of the mRNA expression level of *Bax* and *BCl2* (*n* = 4 per group). (J) Western blot analysis (top) and quantitative results (bottom) of apoptosis‐related proteins (Bax, C‐Caspase3 and Bcl2) in brain tissues from AAV‐Cstb and AAV‐Vector mice following t/MCAO (*n* = 3 per group). Data are presented as the mean ± SD. *p < 0.05, **p < 0.01.

To determine whether CSTB overexpression confers protection against ischemic stroke, we adopted a gain‐of‐function strategy by generating CSTB‐overexpressing mice (AAV‐Cstb), using AAV‐empty vector‐injected mice (AAV‐vector) as controls. Elevated CSTB protein levels were confirmed via Western blot (Figure 3A). After t/MCAO, CSTB‐overexpressing mice exhibited improved neurological scores (Figure 3B), significantly reduced cerebral infarct volume (Figure 3C), attenuated histological damage (Figure 3D), and suppressed neuroinflammation (Figure 3E–G) compared with controls. Furthermore, CSTB overexpression led to decreased TUNEL‐positive cells, reduced the expression of pro‐apoptotic proteins Bax and C‐Caspase 3, and increased the levels of anti‐apoptotic Bcl2 (Figure 3H–J). In contrast to the detrimental effects of CSTB knockdown, these findings indicate that CSTB overexpression is associated with significant protection in the CIRI mouse model.

### 3.4 CSTB Protects Neurons Against OGD/R‐Induced Neuronal Injury

**FIGURE 4:**
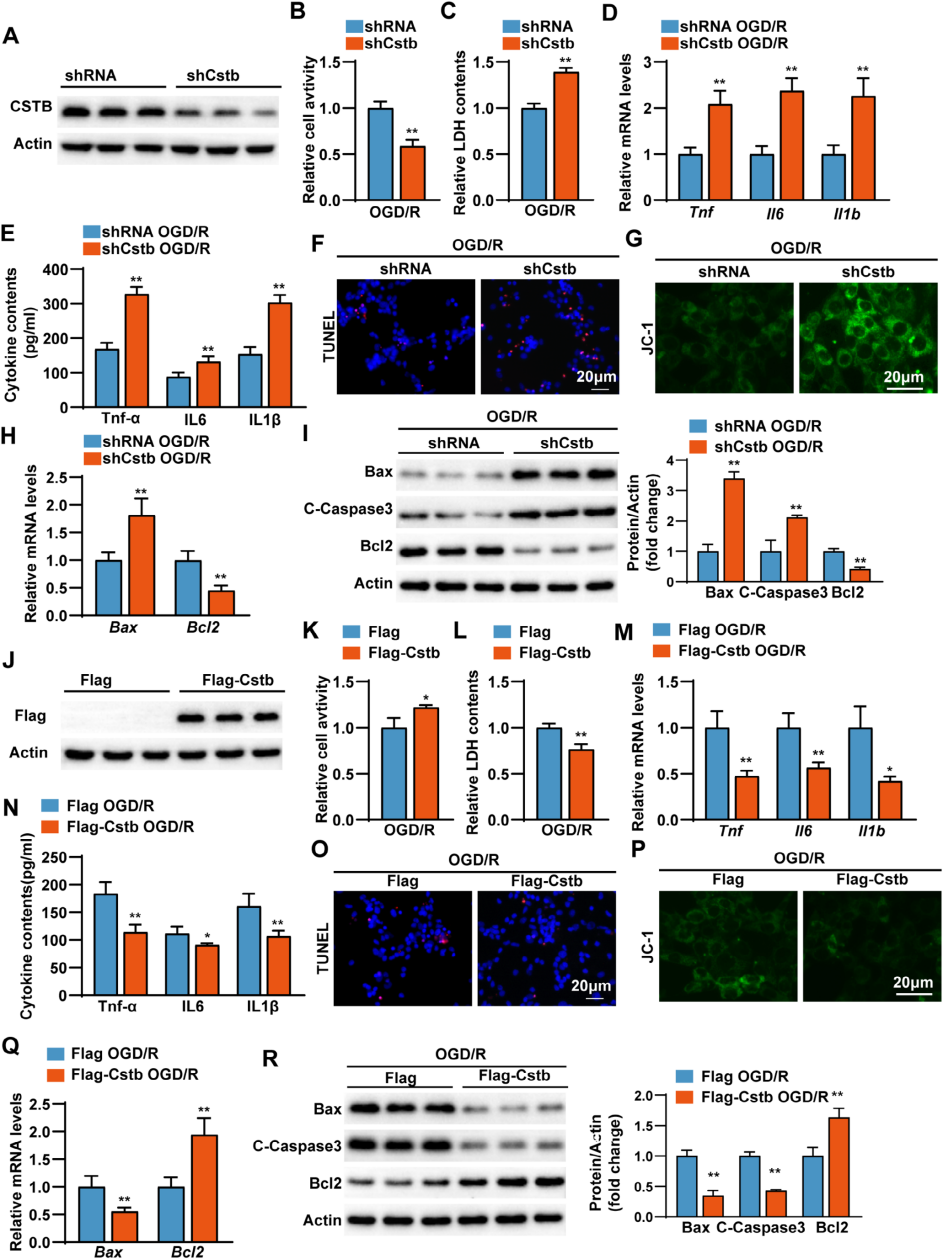
CSTB protects HT22 neurons against OGD/R‐induced neuronal injury. (A) Identification of *Cstb* knockdown in HT22 neurons by Western blot analysis. (B) Cell viability of *Cstb‐*knockdown (shCstb) and control (shRNA) cells after OGD/R. (C) LDH contents of shCstb and shRNA cells after OGD/R. (D, E) The mRNA (D) and protein (E) levels of pro‐inflammatory cytokines in the culture medium of shCstb and shRNA cells following OGD/R. (F) Representative images of TUNEL (red) and DAPI (blue) staining of shCstb and shRNA cells after OGD/R. Scale bar, 20 μm. (G) Representative images of JC‐1 staining of shCstb and shRNA cells after OGD/R. Scale bar, 20 μm. (H) Q‐PCR analysis results of the mRNA expression level of *Bax* and *BCl2* from shCstb and shRNA cells after OGD/R. (I) Western blot (left) and quantification (right) results of Bax, C‐Caspase3 and Bcl2 from shCstb and shRNA cells after OGD/R. (J) Confirmation of successful *Cstb* overexpression in HT22 neurons via Western blotting. (K) Cell viability of *Cstb*‐overexpression (Flag‐Cstb) and control (Flag) cells after OGD/R. (L) LDH contents of Flag‐Cstb and Flag cells after OGD/R. (M–N) The mRNA (M) and protein (N) levels of pro‐inflammatory cytokines from Flag‐Cstb and Flag cells after OGD/R. (O) Representative images of TUNEL (red) and DAPI (blue) staining from Flag‐Cstb and Flag cells after OGD/R. Scale bar, 20 μm. (P) Representative images of JC‐1 staining from Flag‐Cstb and Flag cells after OGD/R. Scale bar, 20 μm. (Q) Q‐PCR analysis results of the mRNA expression level of *Bax* and *BCl2* from Flag‐Cstb and Flag cells after OGD/R. (R) Western blot (left) and quantification (right) results of Bax, C‐Caspase3 and Bcl2 from Flag‐Cstb and Flag cells after OGD/R. *n* = 3–4 independent experiments. Data are presented as the mean ± SD. *p < 0.05, **p < 0.01.

To elucidate the role of CSTB in neurons, which are critical for brain function [34, 35], we knocked down *Cstb* in HT22 neurons using a lentiviral vector encoding *Cstb*‐specific shRNA (shCstb), with a scrambled shRNA as the control. Knockdown efficiency was confirmed by Western blot (Figure 4A). CCK‐8 and LDH assays revealed that *Cstb* knockdown significantly decreased cell viability and increased LDH release after OGD/R (Figure 4B,C), suggesting enhanced neuronal injury. We further examined the effects of *Cstb* knockdown on inflammation and apoptosis. Q‐PCR and ELISA showed that *Cstb* knockdown markedly increased mRNA and protein levels of pro‐inflammatory cytokines induced by OGD/R (Figure 4D,E). Additionally, TUNEL staining indicated a higher number of apoptotic cells in the knockdown group after OGD/R stimulation (Figure 4F). Furthermore, JC‐1 staining to assess mitochondrial membrane potential changes showed that *Cstb* knockdown was linked to impaired mitochondrial function, thereby promoting apoptosis (Figure 4G). Consistently, Q‐PCR and Western blot analysis demonstrated that *Cstb* knockdown upregulated pro‐apoptotic proteins (Bax and C‐Caspase 3) and downregulated anti‐apoptotic Bcl2 (Figure 4H,I). Subsequently, we established HT22 neuronal cells stably overexpressing *Cstb* (Figure 4J) and subjected them to OGD/R stimulation. In contrast to knockdown, *Cstb* overexpression attenuated OGD/R‐induced neuronal damage, as well as the inflammatory response and apoptosis (Figure 4K–R).

To further validate the regulatory role of CSTB in primary neuronal function, we isolated primary neurons from neonatal rats and infected them with a *Cstb* knockdown adenovirus (Ad‐shCstb) to suppress *Cstb* expression (Figure S1A). Consistent with the results observed in HT22 cells, *Cstb* knockdown in primary neurons also exacerbated OGD/R‐induced neuronal damage, inflammatory response, and apoptosis (Figure S1B–I). Furthermore, primary neurons were infected with a *Cstb* overexpression adenovirus (Ad‐Cstb) followed by OGD/R stimulation. Similarly, we demonstrated that Cstb overexpression attenuated OGD/R‐induced neuronal damage, inflammatory activation, and apoptosis (Figure S1J–R). Collectively, these results suggest that CSTB confers protection against neuronal damage, inflammation, and apoptosis during CIRI.

### 3.5 CSTB Inhibits the Activation of JAK2/STAT3 Signaling During CIRI

**FIGURE 5:**
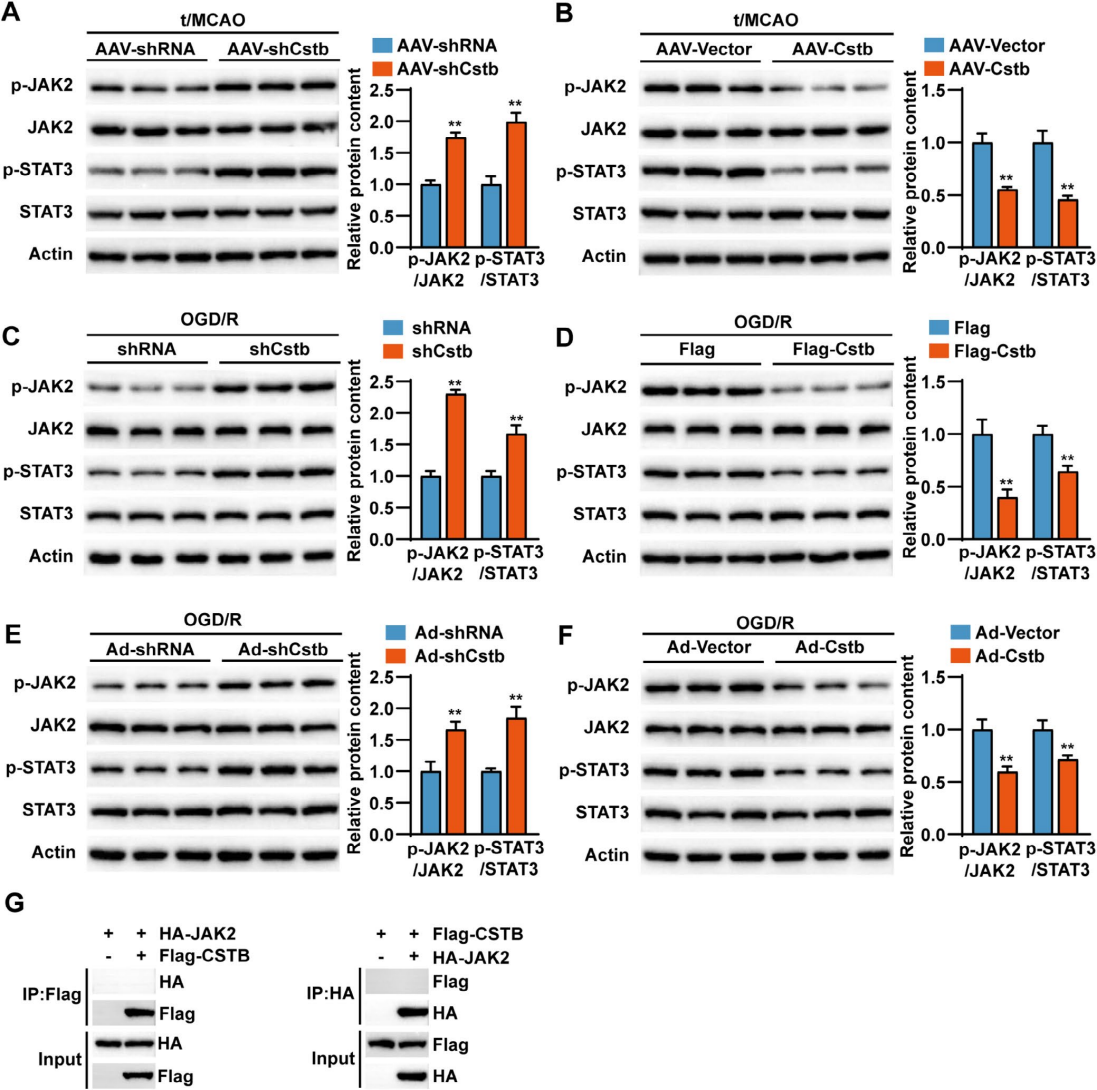
CSTB inhibits the activation of JAK2/STAT3 signaling. (A) Western blot (left) and quantification (right) results of key JAK2/STAT3 pathway proteins (p‐JAK2, JAK2, p‐STAT3, and STAT3) in brain tissues from *Cstb*‐knockdown (AAV‐shCstb) and control (AAV‐shRNA) mice following t/MCAO (*n* = 3 per group). (B) Western blot (left) and quantification (right) results of p‐JAK2, JAK2, p‐STAT3, and STAT3 in brain tissues from *Cstb*‐overexpression (AAV‐Cstb) and control (AAV‐Vector) mice following t/MCAO (*n* = 3 per group). (C) Western blot (left) and quantification (right) results of key JAK2/STAT3 signaling components (p‐JAK2, JAK2, p‐STAT3, and STAT3) from *Cstb*‐knockdown (shCstb) and control (shRNA) HT22 neurons after OGD/R. (D) Western blot (left) and quantification (right) results of p‐JAK2, JAK2, p‐STAT3, and STAT3 from *Cstb*‐overexpression (Flag‐Cstb) and control (Flag) HT22 neurons post‐OGD/R. (E) Western blot (left) and quantification (right) results of key JAK2/STAT3 pathway proteins (p‐JAK2, JAK2, p‐STAT3, and STAT3) after OGD/R in *Cstb*‐knockdown (Ad‐Cstb) and control (Ad‐shRNA) rat primary neurons. (F) Western blot (left) and quantification (right) results of p‐JAK2, JAK2, p‐STAT3, and STAT3 after OGD/R in *Cstb*‐overexpression (Ad‐Cstb) and control (Ad‐Vector) rat primary neurons. (G) Co‐IP with antibodies against Flag (left) and HA (right). For C–G, *n* = 3 independent experiments. Data are presented as the mean ± SD. *p < 0.05, **p < 0.01.

The Janus kinase 2 (JAK2)/signal transducer and activator of transcription 3 (STAT3) signaling pathway modulates critical neuronal processes—including growth, immune responses, and cell death—and is strongly implicated in CIRI [36, 37]. To identify downstream pathways involved in CSTB function, we examined the expression of p‐JAK2, JAK2, p‐STAT3, and STAT3 under both in vivo and in vitro conditions of CSTB modulation. These proteins were analyzed in vivo in Cstb‐knockdown and Cstb‐overexpressing mice subjected to t/MCAO (Figure 5A,B). Analyses were also performed in vitro using OGD/R‐stimulated HT22 cells (Figure 5C,D) and rat primary neurons (Figure 5E,F) following *Cstb* knockdown or overexpression. Both in vivo and in vitro results consistently showed that p‐JAK2 and p‐STAT3 levels were markedly elevated upon CSTB knockdown, but reduced under CSTB overexpression. These findings suggest that CSTB negatively regulates the activation of the JAK2/STAT3 signaling pathway during CIRI. Further Co‐IP assays were conducted to examine the interaction between CSTB and JAK2. The results indicated no direct interaction between CSTB and JAK2, suggesting that CSTB likely influences JAK2 indirectly (Figure 5G).

### 3.6 JAK2/STAT3 Pathway Inhibition Reverses the Promotion Effects of CSTB Knockdown in CIRI

**FIGURE 6:**
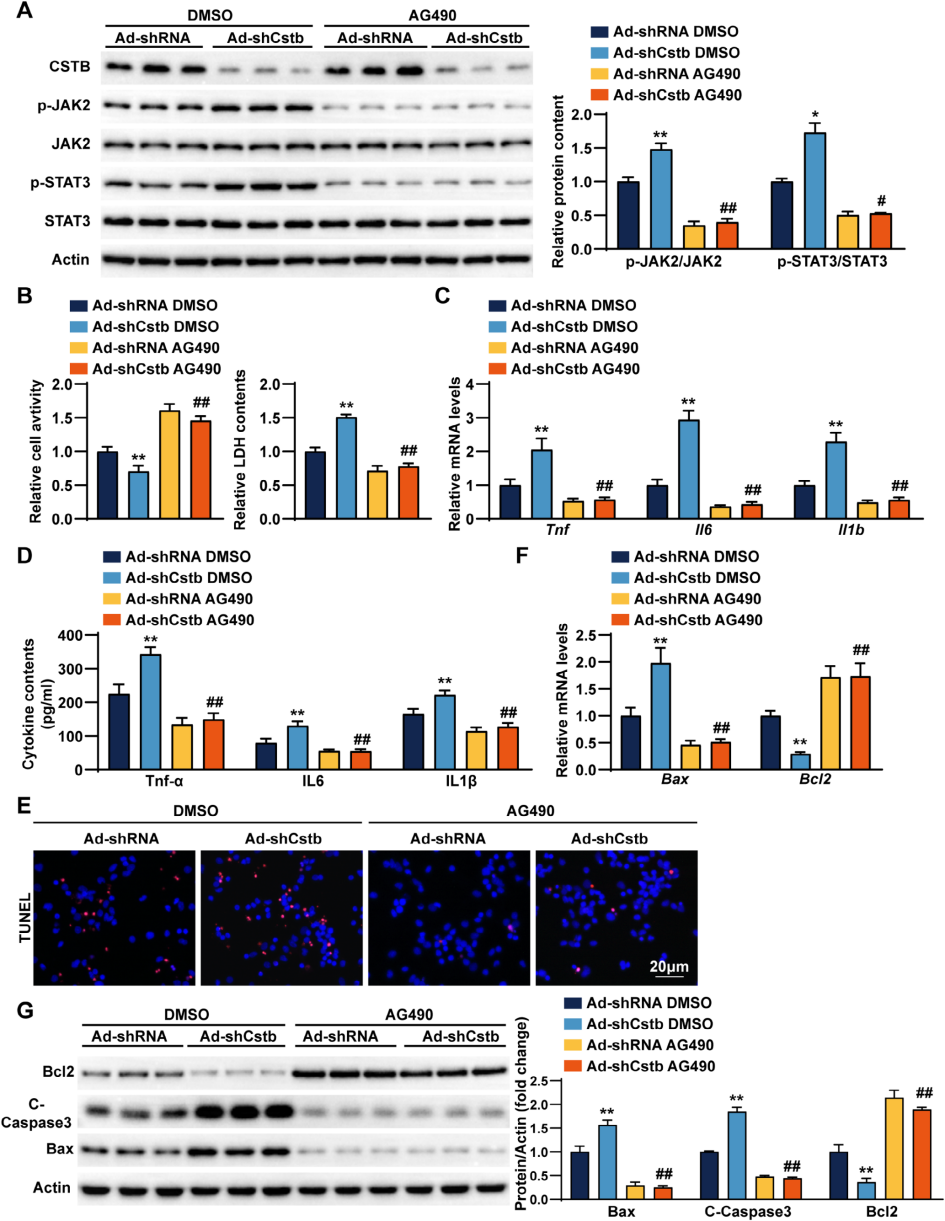
JAK2/STAT3 pathway inhibition reverses the promotion effects of CSTB knockdown in CIRI. (A) Western blot (left) and quantification (right) of CSTB and JAK2/STAT3 pathway protein expression after OGD/R in *Cstb*‐knockdown (Ad‐Cstb) and control (Ad‐shRNA) rat primary neurons treated with DMSO or AG490. (B) Cell viability (left) and LDH contents (right) after OGD/R in Ad‐Cstb and Ad‐shRNA cells treated with DMSO or AG490. (C, D) Pro‐inflammatory cytokine mRNA (C) and protein (D) levels in the culture medium of Ad‐Cstb and Ad‐shRNA cells after OGD/R, with DMSO or AG490 treatment. (E) Representative images of TUNEL (red) and DAPI (blue) staining after OGD/R in Ad‐Cstb and Ad‐shRNA cells treated with DMSO or AG490. Scale bar, 20 μm. (F) Q‐PCR analysis results of the mRNA expression level of *Bax* and *BCl2* after OGD/R in Ad‐Cstb and Ad‐shRNA cells treated with DMSO or AG490. (G) Western blot (left) and quantification (right) results of Bax, C‐Caspase3 and Bcl2 after OGD/R in Ad‐Cstb and Ad‐shRNA cells treated with DMSO or AG490. *n* = 3–4 independent experiments. Data are presented as the mean ± SD. *p < 0.05, **p < 0.01 indicate significant differences between Ad‐shRNA DMSO group and Ad‐Cstb DMSO group. #*p* < 0.05, ##*p* < 0.01 indicate significant differences between Ad‐shCstb DMSO group and Ad‐shCstb AG490 group.

To determine whether CSTB function is mediated through JAK2/STAT3 activation, we treated *Cstb* knockdown cells with the JAK2 inhibitor AG490 and then subjected them to OGD/R stimulation. Western blot analysis showed that AG490 significantly inhibited the upregulation of p‐JAK2 and p‐STAT3 induced by CSTB knockdown (Figure 6A). CCK‐8 and LDH assays revealed that decreased cell viability in Ad‐shCstb neurons was markedly restored by AG490 treatment (Figure 6B). Furthermore, AG490 abolished the upregulation of inflammatory cytokines at both mRNA and protein levels resulting from CSTB knockdown (Figure 6C,D). Similarly, AG490 counteracted the pro‐apoptotic effects of CSTB knockdown, as shown by altered expression of apoptosis‐related molecules (Figure 6E–G). The experimental results in JAK2‐knockdown cells (AdshJAK2) were consistent with the aforementioned findings (Figure S2). Collectively, these results suggest that the neuroprotective effect of CSTB against CIRI is associated with the inhibition of the JAK2/STAT3 signaling pathway.

## 4 Discussion

This study provides evidence supporting CSTB as a critical endogenous neuroprotective factor in CIRI. The core discovery reveals that CSTB is significantly upregulated in both in vivo and in vitro models of CIRI. Functional experiments employing both knockdown and overexpression consistently confirm the potent protective role of CSTB, as shown by reduced neurological deficits, infarct volume, neuroinflammation, and apoptosis. Importantly, we further demonstrate that CSTB exerts its protection by negatively regulating the activation of the JAK2/STAT3 signaling pathway. Knockdown of CSTB enhanced JAK2 and STAT3 phosphorylation, whereas CSTB overexpression suppressed this pathway. Crucially, both pharmacological inhibition (AG490) and genetic knockdown of JAK2 reversed the detrimental effects associated with CSTB deficiency, confirming the essential involvement of the JAK2/STAT3 pathway in mediating the protective effects of CSTB against CIRI.

With the advancement of investigative efforts into ischemic stroke, a growing number of regulatory molecules have been characterized within its pathological framework [38, 39, 40]. The molecular pathogenesis underlying CIRI has garnered growing research interest, underscoring its potential for revealing novel therapeutic targets. The JAK2/STAT3 signaling pathway serves as a critical regulator in CIRI, modulating key pathological processes including neuroinflammation, apoptosis, oxidative stress, and autophagy [41, 42, 43]. Previous studies have indicated that CSTB participates in multiple signaling pathways and regulatory networks within the nervous system, including the modulation of apoptosis, inflammatory responses, and oxidative stress‐related signaling [44, 45, 46]. This study proposes a novel mechanistic framework in which CIRI induces CSTB expression, leading to suppression of JAK2/STAT3 over‐phosphorylation, which in turn attenuates neuroinflammation and neuronal apoptosis, ultimately improving neurological outcomes.

While our study indictates that CSTB attenuates the JAK2/STAT3 pathway to exert neuroprotection in CIRI Co‐IP results revealed no direct physical interaction between CSTB and JAK2. This suggests an indirect regulatory mechanism, which was further supported by both pharmacological inhibition and genetic knockdown of JAK2. Based on CSTB's function as a cysteine protease inhibitor and the documented role of cathepsins in cleaving signaling molecules [47, 48], we propose that it may modulates JAK2/STAT3 signaling through an indirect, protease‐mediated mechanism. Importantly, this work establishes a functional link between CSTB and the JAK2/STAT3 signaling pathway, offering a fresh perspective on the molecular mechanisms of CIRI. By revealing CSTB as a key regulatory molecule within this pathway, our findings not only deepen the pathophysiological understanding of CIRI but also highlight promising new avenues for therapeutic intervention.

Despite the novel insights into the CSTB‐JAK2/STAT3 axis in CIRI, several limitations of this study should be noted. The specific roles of CSTB within distinct cell types, such as microglia and astrocytes, require further investigation through conditional knockout mouse models to validate its cell‐type specificity. Additionally, the absence of a direct interaction opens intriguing questions about the precise mechanisms by which CSTB influences JAK2/STAT3 activity, which remains a key focus for future research. Future studies should aim to clarify the direct molecular interactions between CSTB and JAK2/STAT3 components, as well as explore CSTB's therapeutic potential, such as the efficacy of administering recombinant CSTB protein or its agonists in the post‐stroke recovery phase.

## 5 Conclusions

In conclusion, our study provides evidence supporting CSTB as a key endogenous neuroprotective factor against CIRI. We further demonstrate that the neuroprotective effect of CSTB is associated with the suppression of the JAK2/STAT3 signaling pathway, revealing a potential therapeutic target for ischemic stroke. Targeting this CSTB–JAK2/STAT3 axis may provide new strategies to ameliorate brain injury and improve functional recovery after stroke.

## Author Contributions

G.X., F.W., and J.W. designed and supervised this research. G.Z., J.G., L.W., and X.W. conducted experiments; G.Z., Y.T., and P.Z. performed statistical analysis; F.H. and G.Z. wrote and edited the manuscript. F.H., G.Z., and P.Z. verified the data. All authors reviewed the final version of the manuscript and provided their approval for its submission.

## Funding

This work was supported by Hubei Provincial Natural Science Foundation of China, 2025AFD324.

## Ethics Statement

All animal experiments were conducted in accordance with relevant guidelines and regulations and were approved by the Animal Ethics Committee of Wuhan Luobin Life Science & Technology Co. Ltd. (Approval No. LBSM2025025).

## Consent

The authors have nothing to report.

## Conflicts of Interest

The authors declare no conflicts of interest.

## Supporting information


**Table S1:** The primer sequences were used in Q‐PCR.
**Table S2:** The antibodies were used in Western blot.
**Figure S1:** CSTB attenuates OGD/R‐induced neuronal injury in rat primary neurons. (A) Confirmation of successful *Cstb* knockdown in rat primary neurons by Western blot. (B) Cell viability after OGD/R in *Cstb‐*knockdown (Ad‐shCstb) and control (Ad‐shRNA) cells. (C) LDH contents after OGD/R in Ad‐shCstb and Ad‐shRNA cells. (D, E) The mRNA (D) and protein (E) levels of pro‐inflammatory cytokines in the culture medium of Ad‐shCstb and Ad‐shRNA cells after OGD/R. (F) Representative images of TUNEL (red) and DAPI (blue) staining after OGD/R in Ad‐shCstb and Ad‐shRNA cells. Scale bar, 20 μm. (G) Representative images of JC‐1 staining after OGD/R in Ad‐shCstb and Ad‐shRNA cells. Scale bar, 20 μm. (H) Q‐PCR analysis results of the mRNA expression level of *Bax* and *BCl2* from Ad‐shCstb and Ad‐shRNA cells after OGD/R. (I) Western blot (left) and quantification (right) results of Bax, C‐Caspase3 and Bcl2 from Ad‐shCstb and Ad‐shRNA cells after OGD/R. (J) Validation of *Cstb* overexpression in rat primary neurons via Western blotting. (K) Cell viability after OGD/R in *Cstb*‐overexpression (Ad‐Cstb) and control (Ad‐Vector) cells. (L) LDH contents after OGD/R in Ad‐Cstb and Ad‐Vector cells. (M–N) The mRNA (M) and protein (N) levels of pro‐inflammatory cytokines from Ad‐Cstb and Ad‐Vector cells after OGD/R. (O) Representative images of TUNEL (red) and DAPI (blue) staining from Ad‐Cstb and Ad‐Vector cells after OGD/R. Scale bar, 20 μm. (P) Representative images of JC‐1 staining from Ad‐Cstb and Ad‐Vector cells after OGD/R. Scale bar, 20 μm. (Q) Q‐PCR analysis results of the mRNA expression level of *Bax* and *BCl2* from Ad‐Cstb and Ad‐Vector cells after OGD/R. (R) Western blot (left) and quantification (right) results of Bax, C‐Caspase3 and Bcl2 from Ad‐Cstb and Ad‐Vector cells after OGD/R. *n* = 3–4 independent experiments. Data are presented as the mean ± SD. *p < 0.05, **p < 0.01.
**Figure S2:** JAK2 knockdown reverses the promotion effects of CSTB knockdown in CIRI. (A) Western blot (left) and quantification (right) of CSTB and JAK2/STAT3 pathway protein expression after OGD/R. (B) Cell viability (left) and LDH contents (right) after OGD/R. (C, D) Pro‐inflammatory cytokine mRNA (C) and protein (D) levels in the culture medium cells after OGD/R. (E) Q‐PCR analysis results of the mRNA expression level of Bax and BCl2 after OGD/R. (G) Western blot (left) and quantification (right) results of Bax, C‐Caspase3 and Bcl2. *n* = 3–4 independent experiments. Data are presented as the mean ± SD. *p < 0.05, **p < 0.01 indicate significant differences between Ad‐shRNA group and Ad‐Cstb group. #*p* < 0.05, ##*p* < 0.01 indicate significant differences between Ad‐shCstb group and Ad‐shCstb + Ad‐shJAK2 group.

## Data Availability

The data that support the findings of this study are available from the corresponding author upon reasonable request.
