# Supplementary material for: Cystatin B Attenuates Cerebral Ischemia Reperfusion Injury by Inhibiting the JAK2/STAT3 Signaling Pathway
Source: CNS Neurosci Ther. 2026 Mar 29;32(4):e70818. doi: 10.1002/cns.70818 (PMC13140931; doi:10.1002/cns.70818)
Supplement: Supplementary file 1 — Table S1: The primer sequences were used in Q‐PCR. Table S2: The antibodies were used in Western blot. Figure S1: CSTB attenuates OGD/R‐induced neuronal injury in rat primary neurons. (A) Confirmation of successful Cstb knockdown in rat primary neurons by Western blot. (B) Cell viability after OGD/R in Cstb‐knockdown (Ad‐shCstb) and control (Ad‐shRNA) cells. (C) LDH contents after OGD/R in Ad‐shCstb and Ad‐shRNA cells. (D, E) The mRNA (D) and protein (E) levels of pro‐inflammatory cytokines in the culture medium of Ad‐shCstb and Ad‐shRNA cells after OGD/R. (F) Representative images of TUNEL (red) and DAPI (blue) staining after OGD/R in Ad‐shCstb and Ad‐shRNA cells. Scale bar, 20 μm. (G) Representative images of JC‐1 staining after OGD/R in Ad‐shCstb and Ad‐shRNA cells. Scale bar, 20 μm. (H) Q‐PCR analysis results of the mRNA expression level of Bax and BCl2 from Ad‐shCstb and Ad‐shRNA cells after OGD/R. (I) Western blot (left) and quantification (right) results of Bax, C‐Caspase3 and Bcl2 from Ad‐shCstb and Ad‐shRNA cells after OGD/R. (J) Validation of Cstb overexpression in rat primary neurons via Western blotting. (K) Cell viability after OGD/R in Cstb‐overexpression (Ad‐Cstb) and control (Ad‐Vector) cells. (L) LDH contents after OGD/R in Ad‐Cstb and Ad‐Vector cells. (M–N) The mRNA (M) and protein (N) levels of pro‐inflammatory cytokines from Ad‐Cstb and Ad‐Vector cells after OGD/R. (O) Representative images of TUNEL (red) and DAPI (blue) staining from Ad‐Cstb and Ad‐Vector cells after OGD/R. Scale bar, 20 μm. (P) Representative images of JC‐1 staining from Ad‐Cstb and Ad‐Vector cells after OGD/R. Scale bar, 20 μm. (Q) Q‐PCR analysis results of the mRNA expression level of Bax and BCl2 from Ad‐Cstb and Ad‐Vector cells after OGD/R. (R) Western blot (left) and quantification (right) results of Bax, C‐Caspase3 and Bcl2 from Ad‐Cstb and Ad‐Vector cells after OGD/R. n = 3–4 independent experiments. Data are presented as the mean ± SD. *p < 0.05, **p < 0.01. Figure S2: [file CNS-32-e70818-s001.docx]

**Supplementary Materials**

**Supplementary Table 1.** The primer sequences were used in Q-PCR.

**Table 1. The primers for Q-PCR.**

| **Gene names** | **Species** | **Forward (5’-3’)** | **Reverse (3’-5’)** |
| --- | --- | --- | --- |
| CSTB | Mice | AGGTGAAGTCCCAGCTTGAAT | GTCTGATAGGAAGACAGGGTCA |
| Il6 | Mice | TAGTCCTTCCTACCCCAATTTCC | TTGGTCCTTAGCCACTCCTTC |
| Il1b | Mice | GCAACTGTTCCTGAACTCAACT | ATCTTTTGGGGTCCGTCAACT |
| Ccl2 | Mice | TACAAGAGGATCACCAGCAGC | ACCTTAGGGCAGATGCAGTT |
| Cxcl1 | Mice | CATGGCTGGGATTCACCTCA | CCTCGCGACCATTCTTGAGT |
| β-actin | Mice | GTGACGTTGACATCCGTAAAGA | GCCGGACTCATCGTACTCC |
| Bax | Mice | TGAGCGAGTGTCTCCGGCGAAT | GCACTTTAGTGCACAGGGCCTTG |
| Bcl2 | Mice | CAACAGGGAGATGTCACCCC | TCAAACAGAGGTCGCATGCT |
| CSTB | Rat | GAGATCGCCGACAAGGTGA | AGGGGTTCAAACACCCTCAAG |
| Tnf | Rat | ATGGGCTCCCTCTCATCAGT | GCTTGGTGGTTTGCTACGAC |
| Il6 | Rat | CCCAACTTCCAATGCTCTCCT | TAGCACACTAGGTTTGCCGA |
| Il1b | Rat | GACTTCACCATGGAACCCGT | CAGGGAGGGAAACACACGTT |
| β-actin | Rat | CCGCGAGTACAACCTTCTTG | TGACCCATACCCACCATCAC |
| Bax | Rat | AGGACGCATCCACCAAGAAG | CAGTTGAAGTTGCCGTCTGC |
| Bcl2 | Rat | CTGGTGGACAACATCGCTCT | GCATGCTGGGGCCATATAGT |

**Supplementary Table 2.** The antibodies were used in Western blot.

**Table 2. The antibodies for Western blotting**

| **Antibody** | **Catalogue number** | **Manufacturer** |
| --- | --- | --- |
| β-actin | HUABIO | EM21002 |
| CSTB | HUABIO | HA720084 |
| p-Ikkβ | CST | 2697 |
| Ikkβ | Abclonal | A2087 |
| IkBα | Abclonal | A19714 |
| p-p65 | Abclonal | AP1294 |
| P65 | Abclonal | A19653 |
| Bax | Abclonal | A19684 |
| C-Caspase3 | CST | 9664 |
| Bcl2 | Abclonal | A19693 |
| p-JAK2 | CST | 3771 |
| JAK2 | Abclonal | A7694 |
| p-STAT3 | Abclonal | AP0705 |
| STAT3 | Abclonal | A16975 |
| Flag | MBL | M185-3L |

**Supplementary Figure 1**


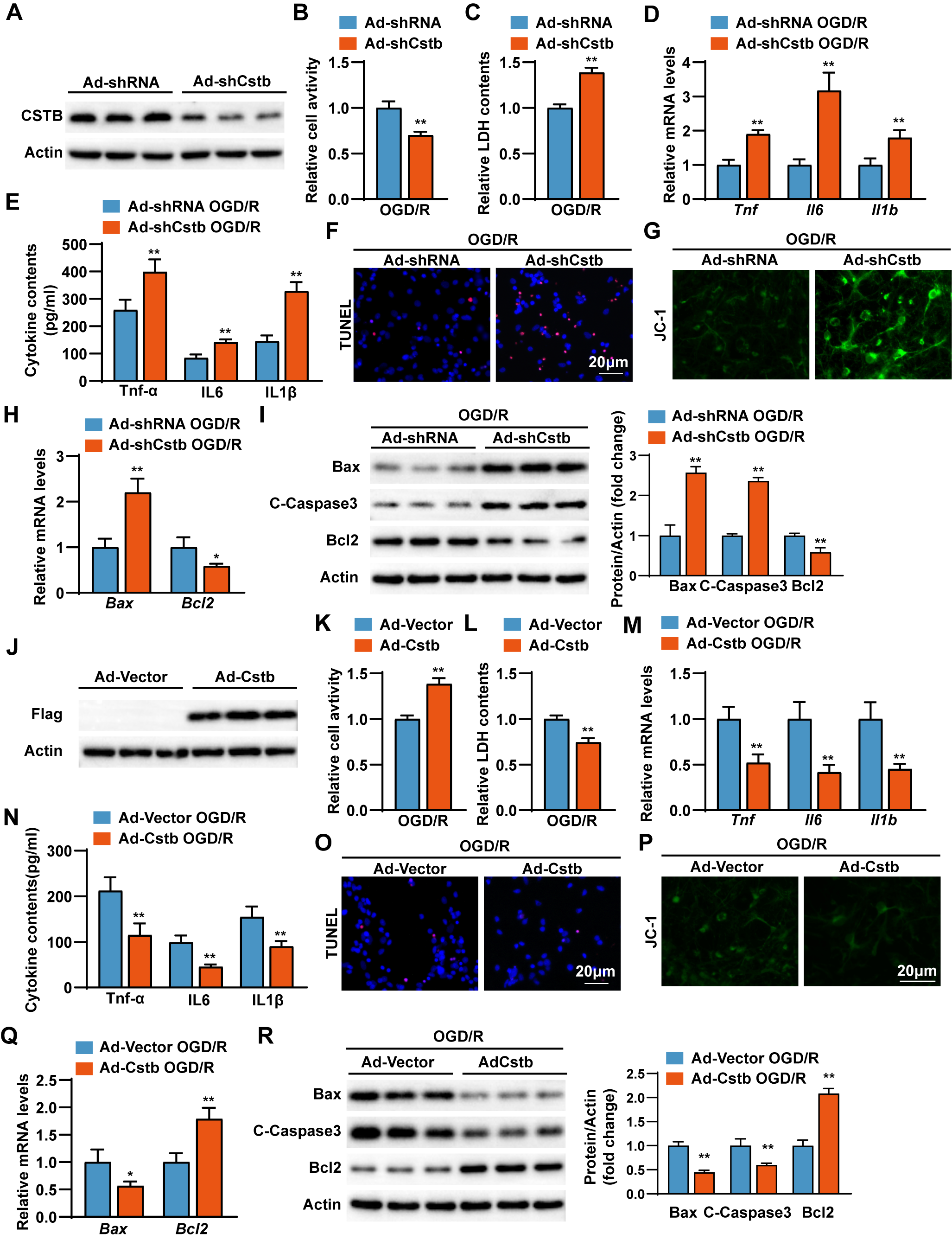


**Supplementary Figure 1. CSTB attenuates OGD/R-induced neuronal injury in rat primary neurons.** (A) Confirmation of successful *Cstb* knockdown in rat primary neurons by Western blot. (B) Cell viability after OGD/R in *Cstb-*knockdown (Ad-shCstb) and control (Ad-shRNA) cells. (C) LDH contents after OGD/R in Ad-shCstb and Ad-shRNA cells. (D–E) The mRNA (D) and protein (E) levels of pro-inflammatory cytokines in the culture medium of Ad-shCstb and Ad-shRNA cells after OGD/R. (F) Representative images of TUNEL (red) and DAPI (blue) staining after OGD/R in Ad-shCstb and Ad-shRNA cells. Scale bar, 20 μm. (G) Representative images of JC-1 staining after OGD/R in Ad-shCstb and Ad-shRNA cells. Scale bar, 20 μm. (H) Q-PCR analysis results of the mRNA expression level of *Bax* and *BCl2* from Ad-shCstb and Ad-shRNA cells after OGD/R. (I) Western blot (left) and quantification (right) results of Bax, C-Caspase3 and Bcl2 from Ad-shCstb and Ad-shRNA cells after OGD/R. (J) Validation of *Cstb* overexpression in rat primary neurons via Western blotting. (K) Cell viability after OGD/R in *Cstb*-overexpression (Ad-Cstb) and control (Ad-Vector) cells. (L) LDH contents after OGD/R in Ad-Cstb and Ad-Vector cells. (M–N) The mRNA (M) and protein (N) levels of pro-inflammatory cytokines from Ad-Cstb and Ad-Vector cells after OGD/R. (O) Representative images of TUNEL (red) and DAPI (blue) staining from Ad-Cstb and Ad-Vector cells after OGD/R. Scale bar, 20 μm. (P) Representative images of JC-1 staining from Ad-Cstb and Ad-Vector cells after OGD/R. Scale bar, 20 μm. (Q) Q-PCR analysis results of the mRNA expression level of *Bax* and *BCl2* from Ad-Cstb and Ad-Vector cells after OGD/R. (R) Western blot (left) and quantification (right) results of Bax, C-Caspase3 and Bcl2 from Ad-Cstb and Ad-Vector cells after OGD/R. n = 3–4 independent experiments. Data are presented as the mean ± SD. * *P* < 0.05, ** *P* < 0.01.

**Supplementary Figure 2**


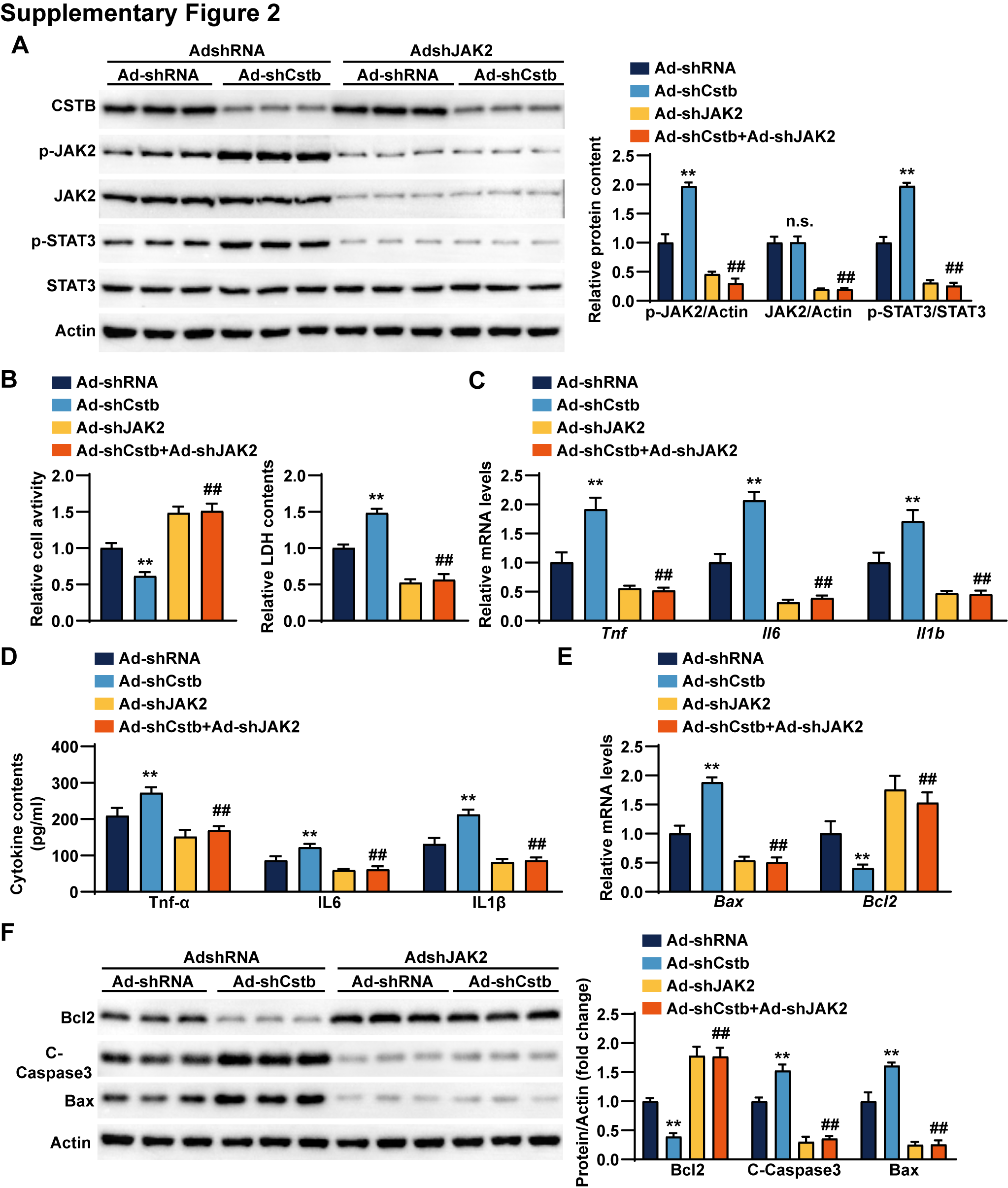


**Supplementary Figure 2. JAK2 knockdown reverses the promotion effects of CSTB knockdown in CIRI.** (A) Western blot (left) and quantification (right) of CSTB and JAK2/STAT3 pathway protein expression after OGD/R. (B) Cell viability (left) and LDH contents (right) after OGD/R. (C–D) Pro-inflammatory cytokine mRNA (C) and protein (D) levels in the culture medium cells after OGD/R. (E) Q-PCR analysis results of the mRNA expression level of Bax and BCl2 after OGD/R. (G) Western blot (left) and quantification (right) results of Bax, C-Caspase3 and Bcl2 . n = 3–4 independent experiments. Data are presented as the mean ± SD. * *P* < 0.05, ** *P* < 0.01 indicate significant differences between Ad-shRNA group and Ad-Cstb group. # *P* < 0.05, ## *P* < 0.01 indicate significant differences between Ad-shCstb group and Ad-shCstb + Ad-shJAK2 group.
